# Challenges associated with the identification of germline variants on myeloid malignancy genomic profiling—a Singaporean experience

**DOI:** 10.3389/fonc.2023.1182639

**Published:** 2023-10-04

**Authors:** Hui-Lin Chin, Joyce Ching Mei Lam, Dheepa Christopher, Poon Limei Michelle, Benedict Yan Junrong

**Affiliations:** ^1^ Khoo Teck Puat National University Children's Medical Institute, Department of Paediatrics, National University Hospital, Singapore, Singapore; ^2^ Department of Paediatrics, National University of Singapore, Singapore, Singapore; ^3^ Children’s Blood and Cancer Centre, KK Women’s and Children’s Hospital, Singapore, Singapore; ^4^ Duke-National University of Singapore (NUS) Medical School, Singapore, Singapore; ^5^ Department of Haematology, Tan Tock Seng Hospital, Singapore, Singapore; ^6^ Department of Laboratory Medicine, Khoo Teck Puat Hospital, Singapore, Singapore; ^7^ Department of Haematology-Oncology, National University Cancer Institute, National University Hospital, Singapore, Singapore; ^8^ Department of Laboratory Medicine, National University Hospital, Singapore, Singapore

**Keywords:** myeloid neoplasm, genetic counselling, gene variants, germline variants, incidental findings

## Abstract

Genomic profiling to identify myeloid-malignancy-related gene mutations is routinely performed for patients with suspected or definite myeloid malignancies. The most common specimen types in our experience are peripheral blood and bone marrow aspirates. Although primarily intended to identify somatic mutations, not infrequently, potentially clinically significant germline variants are also identified. Confirmation of the germline status of these variants is typically performed by hair follicle or skin fibroblast testing. If the germline variant is classified as a pathogenic or likely pathogenic variant and occurs in a gene known to be associated with a disease relevant to the patient’s phenotype (for example, the identification of a *DDX41* pathogenic variant in an individual with acute myeloid leukemia), the management algorithm is typically quite straightforward. Challenging situations may occur such as when the germline variant is classified as a pathogenic or likely pathogenic variant and occurs in a gene not known to be associated with the patient’s phenotype/presenting complaint. We have encountered several such challenging cases in which potentially clinically significant germline variants were identified on the initial genomic profiling of peripheral blood or bone marrow aspirate. In this article, we present these cases and discuss the genetic counseling and management approaches.

## Introduction

Genetic tests are increasingly routinely being done for patients with suspected or definite myeloid malignancies. Molecular variation, including cytogenetic rearrangements and other genetic variation, is increasingly incorporated in the diagnostic evaluation and definitions of myeloid malignancies (1–3). Genetic tests used in the analysis of suspected myeloid neoplasms include karyotype, fluorescent *in situ* hybridization, Sanger sequencing of single genes, and next-generation sequencing (NGS)-based gene panel analysis (4). Such findings influence the diagnosis and prognostication and inform on specific targeted therapies that the proband could be eligible for.

The use of NGS to assess genetic variation is widely becoming more affordable and accessible, enabling concurrent mutational analysis of many genes. This enables ease of access for diagnostic and prognostic purposes on a larger scale. Typically, blood or bone marrow specimens are sent for analysis. NGS gene panels can be divided into two main types, somatic or germline genetic tests.

## Differences between somatic and germline tests

Somatic genetic tests aim to evaluate for variants typically associated with malignancies. They can be done on genomic DNA isolated from the tumor tissue, bone marrow aspirate, or blood. This is typically performed as part of personalized cancer care for the patient. Such findings influence diagnostic classification, disease prognostication, drug eligibility, and other treatment-related decisions.

Germline tests aim to evaluate for variants predisposing to hereditary cancer syndromes. Familial myeloid malignancies are a recognized disease entity under the 2016 World Health Organization (WHO) classification of hematological cancers (3) and European Leukemia Net (ELN) (2). Cohort studies of AML patients have observed the presence of germline variants in 4%–13% of patients (5–7). Genes that have been implicated with germline associations with hematological malignancies include *RUNX1 (* 8), *DDX41 (* 9), *CEPBA (* 10), *TERT (* 11), *GATA2 (* 12), *ETV6 (* 13), *DNMT3A (* 14) *ANKRD26 (* 15), and RAS-MAPK pathway genes (16). Specific inherited bone marrow failure syndromes such as Schwachman Diamond syndrome, Diamond Blackfan anemia, Fanconi syndrome, and short telomere syndrome may have associated characteristic physical findings or systemic features prompting suspicion (17, 18). Germline tests may be more frequently ordered for individuals with a strong family history of cancer, multiple primary malignancies, early onset of cancer, cancers affecting both paired organs, and association with congenital anomalies (17, 19). The identification of germline variants in higher risk individuals such as those with multiple affected family members can be as high as 57% (20).

## Somatic tests can identify germline variants

Genetic tests designed for somatic variant analysis can identify germline variants, somatic variants, and clonal hematopoietic DNA variation (21). Germline variants have been observed in 21% of such cases (22).

Distinguishing the etiology of each variant can be challenging (21). The gold standard for establishing that a variant is somatic involves paired analysis of both tumor and paired non-neoplastic tissue DNA (23). However, this method is costly, requires routine acquisition of multiple biological samples from the patient, and brings about the need for pre- and post-test germline genetic counseling, hence limiting its uptake (23).

A germline variant can be suspected when the variant is reported with a mean allele frequency (MAF) or variant allele frequency (VAF) close to 50% or 100% (22, 24). The value of 50% or 100% could approximate germline heterozygous or homozygous status, respectively. The MAF or VAF refers to the frequency at which the variant is detected in the specimen. This value is also sometimes used to approximate disease burden. Variants with a low VAF <30% are typically considered to be somatic variants (25). For example, in *DDX41*, germline testing for variants identified on somatic analysis for variants >40% VAF confirmed germline variation in 94% of those tested (26).

Identifying the variant in multiple cell lines of different embryological origin could also lead one to derive that the variant in question is likely germline. The blood and bone marrow are of hematopoietic origin from the mesoderm, skin fibroblasts are from the mesoderm, while hair follicle cells are from the ectoderm. Skin fibroblast testing *via* skin biopsy is considered the gold standard for the evaluation of germline variation (27). In our center, hair follicle testing is generally considered acceptable by patients, while skin fibroblast biopsy has a limited uptake due to its perceived invasiveness. Saliva and buccal swabs contain a mix of blood-derived leukocytes and buccal epithelial cells and have been shown to have a higher tumor content compared to skin fibroblasts (28). Hence, they are less suitable for differentiating germline versus somatic variation (29).

Additionally, the persistence of the variant identified on sequential clinical NGS somatic panels, despite a change in disease state, increases the likelihood that this variant is germline.

The absence of a family history of hematological issues or malignancies does not exclude a germline cause (17). This is because genetic variants can arise *de novo* or be a recessive disorder inherited from asymptomatic carrier parents. Certain heritable cancer predispositions may also have variable expression or penetrance. There may not also be a strong correlation between age of onset and presence of germline predisposition (5).

Complicating our understanding, variants detected on somatic panels have been also linked to clonal hematopoiesis arising with age or as a result of previous chemotherapy and somatic mosaicism (30). There are currently no formal recommendations for the interpretation of such data in the context of clinical care.

Novel methods such as computational analysis of sequence variants derived from the blood or bone marrow would be a cost-effective method for differentiating germline versus somatic variation without the need for further sampling and testing (30, 31). One study developed such an algorithm for solid tumors with high precision accuracy (31). A comparable tool has not yet been established for myeloid neoplasms.

## Confirmation of germline variants

When a variant is suspected to be germline, physicians can consider testing other cell lines for the specific variant unlikely to be affected by the hematological malignancy, such as hair follicle or skin fibroblasts.

Another possible method to clarify such variants could be by testing other family members for the specific variant. The identification of one or more family members with the same rare variant will make it more probable that the variant in question is germline.

## Importance and implication of clarifying whether a variant is germline

It is important to suspect when identified variants could be germline and pursue further evaluation. Such information could be medically important and influence one’s health and management in multiple ways. The American College of Medical Genetics (ACMG) additionally recommends further assessment and reporting actionable incidental findings in a curated list of genes (32). Germline disease-associated variants in genes such as *RUNX1, TP53*, *WT1*, and *PTEN*, which are also commonly assessed on myeloid gene panels, would fall under this category.

1. Treatment, prognostication, and future health surveillance
   Certain germline familial cancer predisposition syndromes influence risk for systemic manifestations and associated conditions. For example, individuals with *TP53* disease variants have an increased lifetime risk for soft tissue sarcomas, breast cancer, brain tumor, leukemias, and other malignancies (33). There are established recommendations for health surveillance, which should be incorporated in the proband’s treatment plan. The underlying syndrome may be associated with other health predispositions, which should be evaluated for, such as *PTPN11*-related Noonan syndrome and structural cardiac abnormalities. Certain findings may influence management-related decisions; for example, individuals with *TP53* disease variants should not be exposed to radiation due to the risk of malignant transformation, influencing treatment planning (34). Genes that are less well understood may not have similarly well-established treatment guidelines.
2. Heritability and family planning
   Germline variants are heritable, i.e., they can be passed to offspring of the proband. Offspring of probands with conditions inherited in an autosomal dominant manner have up to 50% risk of inheriting the variant. Offspring of probands with recessive conditions are obligate carriers. This knowledge may influence family-planning-related decisions for the proband and his family.
3. Donor selection for bone marrow transplant
   In the event that a proband requires a bone marrow transplant or HSCT, allo-identical HLA-matched donors are the first choice to optimize treatment outcomes. These could be siblings, other biological relatives, or unrelated donors sourced from donor registries. However, such individuals could be also at risk of carrying the same familial variant. Using a donor carrying the same genetic predisposition could increase the proband’s risk of post-transplant complications and future malignancy and therefore is not advisable (35).

## Outcomes of genetic testing

Genetic test reports for somatic variation typically contain variants thought to be of clinical significance by the laboratory geneticist. Some laboratories categorize reported findings into one of four tiers depending on the expected clinical significance of the finding (24). Other laboratories utilize the ACMG and Association of Molecular Pathology standards and guidelines for variant interpretation (36), although it is important to note that these were developed for rare disease and modifications for certain criteria may need to be considered for germline variants related to myeloid neoplasms. This categorization depends on available evidence about the reported variant and actionability of the findings.

Somatic variation in genes frequently mutated in myeloid neoplasms and myelodysplastic syndromes can also exist in the blood sample of otherwise healthy individuals due to clonal hematopoiesis (37). These may represent early events in the development of hematological malignancies and therefore future predisposition but may not contribute to a diagnosis at the time of testing.

## Risks of genetic testing

For the clinician, inappropriate testing, inappropriate result interpretation, or inappropriate follow-up action for the findings on genetic tests can result in harm to the patient, his relatives, and be susceptible to potential risks for patient complaints, medicolegal actions, and increased healthcare costs (38).

The risk of identifying a germline variant in an individual may come with certain implications that the provider and patient should be aware of as follows:

1. Implication on insurance claims and medical funding
   The knowledge about whether an individual’s condition is due to a germline predisposition can influence insurance coverage and medical funding for healthcare costs in certain healthcare funding models. The need for obtaining prior authorization and insurance denials may limit the uptake of germline testing,
2. Health surveillance for at-risk family members
   Depending on the mode of inheritance of the variant and gene in question, other biological relatives of the proband, e.g., siblings, offspring, and parents, could also carry the variant. These individuals could be at risk of similar hematological issues and benefit from health surveillance for early detection or pre-emptive treatment. Biological relatives of the proband should be offered genetic counseling and testing for risk assessment and to facilitate personalized medicine for that individual.
3. Psychological stress and other implications
   The knowledge about whether an individual’s condition is due to a germline predisposition can influence feelings of anxiety, stress, depression, and guilt. Fear of heightened anxiety surrounding genetic findings can hinder acceptance of testing (30). The possibility that these could be heritable can also implicate family relationships.
4. Risk of a variant of uncertain significance
   A variant of uncertain significance (VUS) refers to a genetic variant whose impact on health and disease is unknown (24). This is usually due to insufficient evidence in the medical and scientific literature about the effects of the specific finding, although this knowledge can change as more information is accrued in time. This risk is reduced for genes that are more extensively evaluated with gene-specific variant classification guidelines such as *RUNX1 (* 39). Clinicians need to be aware of the possibility of such findings, and patients receive appropriate post-test counseling to avoid errors of attribution.
5. Risk of an incidental finding
   It is possible to uncover potential incidental genetic diagnoses on somatic myeloid gene panels, as some of the genes included encode germline syndromes and predispositions. The patients being tested may not have previously been suspected with such syndromes, and identification of such potential diagnoses was not the primary purpose of the test (40). Such findings can be challenging to deal with and communicate to the patient.
6. Risk of a false negative test result
   Knowledge of what the test done covers, and does not cover, is important in understanding and counseling for the residual risks. For instance, a patient who is inadequately informed that the genetic test done is to assess for somatic findings only may have the false impression that they had been adequately evaluated for hereditary cancer predispositions. Variants in genes contributing to the patient’s pathology may also not be assessed on the panel selected due to test coverage or technical limitations.

## Case 1

A 4-month-old female proband was noted to have an incidental finding of splenomegaly on a routine well baby check by a general pediatrician. She is the third child of non-consanguineous parents. Her parents and siblings are well with no known medical conditions. Her full blood count showed an increase in total white cell count with monocytosis—white blood cells, 18.3 × 10^9^/L (6–18); hemoglobin, 9.9 g/dL (11.1–14.1); platelets, 107 × 10^9^/L (140–440); neutrophils, 6.41 × 10^9^/L (1.00–6.00); monocytes, 3.66 × 10^9^/L (0.2–1.2); blasts, 2%; promyelocytes, 1%; myelocytes, 2%; and metamyelocytes, 1%. Examination of the blood film showed a leukoerythroblastic blood film with monocytosis and blasts seen, suggestive of a myeloproliferative neoplasm. She underwent a bone marrow examination, which showed a hypercellular bone marrow with left-shifted granulocytic hyperplasia, mild eosinophilia, and increased megakaryopoiesis. A number of megakaryocytes were noted to be dysplastic. Immunophenotyping showed 5% myeloblasts with an expanded monocytic component. The karyotype from the bone marrow was 46XX. Based on her clinical features and findings from the peripheral blood and bone marrow, a diagnosis of juvenile myelomonocytic leukemia (JMML) was suspected.

A clinical myeloid neoplasm NGS panel performed from a peripheral blood specimen revealed the finding NM_002834(PTPN11):c.1472C>A;p.Pro491His, a known variant associated with risk for JMML (41), with a VAF of 49%. This variant has been associated in multiple individuals worldwide with Noonan syndrome with ClinVar entry classifying it as pathogenic/likely pathogenic (42). The possibility that this variant is of germline origin was raised, and the child was referred to the clinical geneticist. Genomic DNA from skin fibroblast culture was sent to a clinical laboratory for a Rasopathies and Noonan spectrum disorders gene panel. This demonstrated the presence of the same variant, classified by the laboratory as pathogenic, confirming that this was germline in origin. Both parents and two siblings tested negative for the familial variant, identifying that this variant was likely *de novo* in origin.

Germline disease-associated variation in *PTPN11* is known to result in autosomal dominant Noonan Syndrome and risk for JMML. This finding was crucial in the management of this child, as myeloid proliferations associated with germline *PTPN11* variants are typically benign and expected to regress spontaneously with time; hence, aggressive treatment of this condition with chemotherapy is to be avoided (43). There was indeed spontaneous improvement in the cell counts observed in this patient, with disappearance of peripheral blasts and monocytosis by 9 months of age.

The child also benefited from attaining the diagnosis of Noonan Syndrome and underwent abdominal ultrasound and echocardiography to screen for associated renal and cardiac abnormalities. She will continue to have regular echocardiograms to monitor for the development of hypertrophic cardiomyopathy.

## Case 2

A 33-year-old male proband, previously well, was referred to Hematology for the evaluation of persistent eosinophilia. This was first detected when he presented with fever, chills, and abdominal pain for a few days. He was noted to have deranged liver function tests, alanine transaminase (ALT) of 345 U/L (10–55) and aspartate transaminase (AST) of 77 U/L (20–45), and was treated with intravenous antibiotics for possible cholecystitis/hepatitis. His absolute eosinophil count on admission was 1.7 × 10^9^/L (0.00–0.60) with a total white blood cell count of 17.9 × 10^9^/L (4.0–9.6). He developed a rash several days after presentation, and skin scraping done was positive for *Trichophyton mentagrophytes* tinea infection. He was treated with topical steroids, clotrimazole, and antihistamines for this. Throughout the subsequent weeks, the proband continued to have elevated eosinophil levels, the highest at 11.68 × 10^9^/L (0.00–0.60).

A bone marrow aspirate and trephine biopsy was performed to investigate the eosinophilia. Bone marrow aspirate showed reactive marrow with eosinophilia and no abnormal lymphoid cells. Flow cytometry showed no conclusive evidence of a clonal B lymphoproliferative disorder. Histology showed normocellular marrow with increased eosinophilic precursors. Cytogenetics from the bone marrow was normal, 46XY. A clinical NGS panel for hematological malignancies done on bone marrow aspirate revealed the presence of NM_005188.4(CBL):c.1256G>A, p.Cys419Tyr with a VAF of 50% and classified as likely pathogenic by the reporting clinical laboratory. Subsequent specific variant testing in hair follicles demonstrated the presence of the same *CBL* variant, confirming the germline nature of this finding. This variant has not been previously reported in other individuals with *CBL*-related disease, but is absent in population databases (gnomAD, 1000Genomes, ExAC) and is predicted deleterious by multiple *in silico* tools including PolyPhen, SIFT, Provean score of 0.9827, and CADD score of 30. The variant additionally occurs in the functionally significant RING domain of the CBL protein (44). There is a recent ClinVar entry listing this variant as an uncertain finding (45).

Germline-disease-associated variants in *CBL* are known to result in a Noonan-like syndrome with or without JMML. Other health predispositions reported with germline *CBL* disease variation include immune dysregulation, malignancies like acute myeloid leukemia, rhabdomyosarcoma, and vasculopathy (46). Hypereosinophilia has additionally been associated with *CBL* (47). The proband was examined for physical features of Noonan syndrome, which were not evident. Nonetheless, the applicability of this finding is not excluded as Noonan syndrome can have variable expressivity (46, 48). This variant was thought to be contributory to the proband’s hematological phenotype given the level of evidence and phenotypic concordance. The proband has since received genetic counseling about this genetic finding and its associated risks. The proband remains on follow-up for anticipatory management and surveillance. Familial segregation was offered but declined.

The proband received empirical treatment for the eosinophilia with prednisolone 1 mg/kg per day for 1 week, followed by a tapering dose regime where he was weaned off steroids over the subsequent 3 weeks. The eosinophil count eventually decreased to normal levels approximately 3 weeks after initial presentation.

## Case 3

A 38-year-old male proband, previously well, presented with fatigue and pallor over several months from pancytopenia with reticulocytopenia due to aplastic anemia. There were no apparent triggers or recent viral infections. The proband was the youngest of four siblings from non-consanguineous parents with no family history of hematological problems. At diagnosis, his investigations showed white blood cells of 2.8×10^9^/L (4.0–9.6), hemoglobin of 6.4g/dL (13.6–16.6), platelets of 19×10^9^/L (150–360), neutrophils of 1.2×10^9^/L (1.9–6.6), reticulocytes of 1.5% (0.5–2.3), and lactate dehydrogenase of 453 U/L (270–550). Bone marrow aspirate showed hypocellular marrow with absent megakaryopoiesis with no evidence of blast cells. Flow cytometry for blasts and lymphoma were negative. Bone marrow cytogenetics analysis revealed 46XY. Bone marrow trephine showed hypocellular marrow (20%–25% cellularity) with evidence of hematopoiesis, erythropoiesis, and granulopoiesis. A clinical NGS panel for hematological malignancies done on bone marrow aspirate revealed the presence of NM_016222.4(DDX41*)*:c.245_248dupAGTC, p.(Asn84fs) with a VAF of 47.15% and classified as a Tier II finding (variants with potential clinical significance). This finding prompted referral to a clinical geneticist for further evaluation. Clinical whole genome sequencing from a buccal swab sample showed that he was heterozygous for the same *DDX41* variant, classified by the laboratory as likely pathogenic. Subsequent specific variant testing in hair follicles also demonstrated the presence of the same *DDX41* variant, confirming the germline nature of this finding. This variant has not been previously associated with the disease but is absent in population databases (gnomAD, 1000Genomes, and ExAC) and predicted to result in frameshift and premature truncation of the DDX41 transcript, consistent with the known disease mechanism.

Individuals with disease-associated *DDX41* variants are at risk of familial myelodysplastic syndrome and acute myeloid leukemia (MDS/AML) with elevated risk for myeloid neoplasms, lymphoid neoplasms, adult-onset single- or multiple-lineage cytopenias (including aplastic anemia), and red blood cell macrocytosis (49).

The proband required allogeneic stem cell transplantation (SCT) for the management of his aplastic anemia. The proband’s siblings underwent HLA typing and *DDX41* familial variant testing as part of the transplant work up. Two of his three siblings were found to be HLA matched but tested positive for the *DDX41* familial variant, thus less suitable as donors. Donors heterozygous for *DDX41* variants could increase the recipient’s risk of post-transplant leukemia (50). The third sibling who was not an HLA match tested negative for the *DDX41* familial variant. As there were no suitable familial donors, a matched unrelated donor (MUD) was identified, and the patient completed successful SCT. The siblings carrying the familial *DDX41* variant received genetic counseling about *DDX41*-related risks for myeloid dysplasia or leukemia and were referred to a hematologist for monitoring and follow-up (49).

## Discussion

The above three cases illustrate challenging situations that can arise involving findings on myeloid genetic panels that the authors have encountered in our clinical practice. These include incidental diagnostic findings highlighting an underlying syndrome necessitating further anticipatory management (Cases 1 and 2) and identification of an underlying familial predisposition implicating bone marrow donor selection and surveillance for at-risk family members (Case 3).

## Challenges that can arise with identification of germline variants on myeloid malignancy somatic panels

Clinicians unfamiliar with dealing with germline variation may not be aware of how to identify potential germline candidates and discuss the further evaluation of these with the patient (35). They may not be familiar with sourcing appropriate tests for germline analysis (35). They additionally may not have adequate time to address these in busy time-starved clinics (35). To minimize this, close collaboration between hematologists, hematopathologists, and geneticists can be encouraged. Laboratory geneticists suspicious of potential germline variants can also aid this process by communicating their concerns to the clinicians and recommend appropriate follow-up tests for the patient.

When a suspected germline variant occurs in a gene not associated with proband phenotype, the patient will benefit from reverse deep phenotyping. This involves a detailed clinical examination and sometimes imaging or other laboratory tests to evaluate for features consistent with or refuting the finding. Syndrome presentations can have a phenotypic variation from classic descriptions of the disorder, and clinicians may not be familiar with these (35). If the primary clinician is unfamiliar with the gene or associated condition, referral to a geneticist or appropriate specialist for review could be helpful.

Even when approached about the potential of a germline variant, patients may decline germline testing due to fear of psychological burden of information, implication to insurance, or unwillingness for procedures to obtain samples such as skin biopsy for fibroblast culture. To increase uptake, having appropriate support for potentially distressing findings, alternative sources of healthcare funding such as nationalized healthcare funding, savings schemes or charity funds, and more approachable sampling like hair follicle testing may be useful.

Knowledge about the natural history and lifetime risks of myeloid malignancy and other health concerns in association with germline syndromes is evolving and may not be well established for many genes (35). Apart from health surveillance, pre-emptive treatment may not be available to reduce one’s health risks. This is likely to improve as more data is accrued with time.

If underlying familial predispositions are identified, cascade genetic testing to family members may benefit them. These individuals should receive genetic counseling and offered testing. If found to carry the familial variant, they could benefit from pre-symptomatic surveillance or early treatment if relevant.

Appropriate genetic counseling and contextualization of the findings for the patient and their family are important to minimize risks of psychological distress, errors of attribution, or missing potential familial predispositions and genetic diagnoses.

At present, germline predispositions to myeloid malignancy are largely underrecognized. Only approximately 25% of familial myelodysplastic/myeloid malignancy cases have an identified genetic predisposition (51). There are more susceptibility genes awaiting discovery. Thus, issues addressing uncovering germline predispositions are likely to arise more frequently in clinical practice in the future. Formal guidelines for the evaluation of and health surveillance for germline predispositions in myeloid neoplasms will support standardization of practice and patient care (52).

## Recommendations for counseling for somatic myeloid genetic panels

1. Adequate pre-test counseling and consent

This is important even for somatic gene panels (53). Patients undertaking somatic genetic tests should be aware that there are benefits and risks of undertaking somatic tumor genetic testing (**Table 1**).

**Table 1:** Recommendations for pre-test counseling for somatic myeloid gene panels.

**Benefits** • This test can enable understanding of patient-specific tumor genetic biomarkers. • Knowledge of patient-specific genetic biomarkers can help your physician make a diagnosis. • This information helps your physician make treatment decisions including drug eligibility and targeted therapy. • This information helps your physician with disease stratification and prognostication.	
**Risks/limitations** • Not all known biomarkers may be included in the test selected. • Genetic tests may uncover incidental findings that could have impact on your health. • Genetic variants that you have been born with (germline) may inadvertently be identified in the process of testing. ⚬ If a variant is suspected to be germline, your doctor may recommend more testing to understand this better. ⚬ Understanding whether a variant is germline may be important for your treatment plan and health. ⚬ Understanding whether a variant is germline could have implications on financial planning and risk to biological family members. ⚬ Your doctor will discuss these with you (or refer you to an appropriate specialist to do so) before further confirmatory testing is done.

2. Communication of test results to the patient

The patient needs appropriate post-test counseling to explain how the findings could impact their diagnosis, prognosis, longer term health risks, and risk to family.

3. Suspect when a genetic variant identified on somatic panel could be germline and pursue evaluation.

Appropriate referral to a medical genetic specialist can be considered to aid this process. This can be done in parallel with treatment for the hematological condition, as evaluation can take time and the results could implicate treatment-related decisions. To minimize the need for multiple procedures for the patient, tests can be timed with other planned procedures as well. For example, skin biopsy for fibroblast culture to facilitate genetic testing can be done during bone marrow aspiration or central line/port-a-cath insertion.

## Conclusion

Somatic myeloid gene panels are a useful tool to help with diagnostic evaluation in patients suspected with myeloid malignancies. Such tests can uncover suspicion for incidental underlying germline findings, which warrant further investigation. Physicians ordering such tests need to be aware of such risks and consider appropriate evaluation for their patients.

## Methods

### Hematological malignancies NGS panel

Next-generation sequencing was performed on genomic DNA isolated from whole blood or bone marrow aspirate to detect genetic alterations in 108 genes per clinical protocol at the Department of Laboratory Medicine, Tan Tock Seng Hospital, Singapore.

### Myeloid neoplasm NGS panel

Next-generation sequencing was performed on genomic DNA isolated from whole blood or bone marrow aspirate to detect genetic alterations in 52 genes per clinical protocol at the Department of Laboratory Medicine, National University Hospital, Singapore.

### Rasopathies and Noonan spectrum disorders gene panel

Next-generation sequencing was performed on genomic DNA isolated from skin fibroblasts to detect genetic alterations in 28 genes per clinical protocol at Invitae Corporation, San Francisco, CA, USA.

### Whole genome sequencing

Clinical whole genome sequencing was performed per clinical protocol at Praxis Genomics LLC, Atlanta, GA, USA.

### Hair-follicle-targeted sequencing

Sanger sequencing of the specific variant was performed on genomic DNA isolated from between 20 and 50 hair follicles at Molecular Diagnostics Laboratory, National University Hospital, Singapore.

## Author contributions

BJ, JL, DC and H-LC conceived the study. H-LC took a lead in writing the manuscript. BJ, JL, DC, PM, H-LC contributed to data collection, analysis and manuscript preparation. All authors contributed to the article and approved the submitted version.
